# *pdCIFplotter*: visualizing powder diffraction data in pdCIF format

**DOI:** 10.1107/S1600576722003478

**Published:** 2022-05-25

**Authors:** Matthew R. Rowles

**Affiliations:** a John de Laeter Centre, Curtin University, GPO Box U1987, Perth, WA 6845, Australia

**Keywords:** powder diffraction, data visualization, CIF, pdCIF

## Abstract

A description is given of the program *pdCIFplotter*. This program is used for visualizing powder diffraction data and models published in powder CIF format (pdCIF). In particular, support for the visualization of multi-pattern data sets, such as *in situ* diffraction experiments, is provided by means of stack and surface plots. *pdCIFplotter* is written in Python 3 and can run wherever a compatible runtime is available. *TOPAS* macros for the production of pdCIF files are also presented.

## Abstract 2

A program is described for visualizing powder diffraction data and models published in powder CIF format.

## 1. Introduction

The Crystallographic Information Framework (CIF) (Bernstein *et al.*, 2016 ▸; Hall *et al.*, 1991 ▸) is a human- and machine-readable text-based file format for the exchange of crystallographic information. Originally constructed for single-crystal data, the core CIF dictionary has been extended to include powder diffraction data (Toby, 2006*a* ▸,*b* ▸) – pdCIF – amongst others. All current CIF dictionary definitions are available from the International Union of Crystallography (IUCr, 2021*a* ▸).

There are a variety of tools available for the creation, viewing and editing of CIF files, many of which are listed online (IUCr, 2021*b* ▸). However, there is a dearth of end-user tools that work well with pdCIF, and as a result, the adoption of pdCIF is not as widespread as it should be. Common powder diffraction analysis software packages are often capable of outputting diffraction data and model results, most often Rietveld refinements (Loopstra & Rietveld, 1969 ▸; Rietveld, 1969 ▸), in pdCIF format but are then not able to read in those same files. This lack of interoperability makes studying the data and models by a third party a difficult process without access to the original software and analysis files. One previous software package, *pdCIFplot* (Toby, 2003 ▸), was written to perform this task but is no longer maintained. The IUCr maintain an online tool, *plotCIF* (IUCr, 2021*c* ▸), which provides largely the same capability. Both are limited to showing a single diffraction pattern at a time.

To expand the available pdCIF software tools for the end-user, *pdCIFplotter* has been written to provide an easy-to-use interface for visualizing powder diffraction data and models published in pdCIF format. In particular, support for the visualization of multi-pattern data sets, such as *in situ* data, is provided by means of stack and surface plots.

## 2. pdCIFplotter

### 2.1. Overview

The key concept on visualizing data with *pdCIFplotter* is that all diffraction patterns that are related by some key feature should be contained in a single CIF file. For example, in an *in situ* experiment, powder diffraction data are included in the CIF file in the order in which they were collected, with information about temperature, pressure and collection time, as well as the calculated patterns, *R* factors (Toby, 2006*c* ▸) and refined crystal structures as a result of any Rietveld modelling.

The key pdCIF data names of interest relating to the diffraction intensities are outlined in Table 1 ▸. Other data names of interest are given in Table 2 ▸. In general, the _pd_meas items are directly measured, _pd_proc items are processed in some manner to obtain their values and _pd_calc are calculated by some modelling process. There may be one or more loops in a data block containing diffraction information; for example, one loop may contain the as-collected data and a second may contain the processed and calculated data. The as-collected data may sometimes need to be scaled to take into account, for example, variations in collection time. The intensity values between _pd_proc and _pd_calc data items should be directly comparable.

The correct linking of data blocks is required. Each diffraction pattern and crystal structure is contained in its own data block and must be identified with a _pd_block_id data item. Those containing crystal structure information should be linked to their respective diffraction data with a _pd_block_dif fractogram_id data item. Those containing diffraction data must link to the relevant structure information, if any, using the _pd_phase_block_id data name. Example data sets (Section 4) are given in the supplementary information. In *pdCIFplotter*, the order in which diffraction data appear in the CIF file is considered to have meaning; *i.e.* data are assumed to have been measured in the order in which they appear in the CIF file.

**Figure 1:**
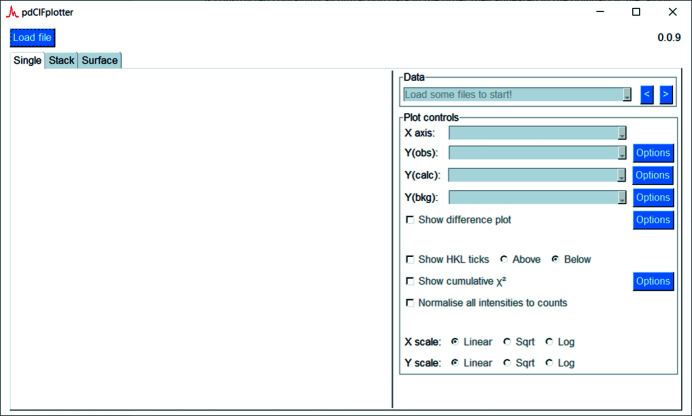
*pdCIFplotter* appearance on first opening.

When *pdCIFplotter* is first opened, the window appears as shown in Fig. 1 ▸. A CIF file can be loaded by pressing the ‘Load file’ button in the top left. The type of data visualization can be chosen by clicking on one of the three tabs, ‘Single’, ‘Stack’ or ‘Surface’, with each visualization displayed in the white-space below the tab. Each visualization can be altered by the controls present in the right panel of the corresponding tab.

When a CIF file is loaded, its contents are parsed with *PyCIFRW* (Hester, 2006 ▸, 2021 ▸), and a dictionary is constructed containing all diffraction data in the order they appeared in the original CIF file, along with relevant metadata. Depending on the *x* coordinates given, and the presence of a valid wavelength, other *x* coordinates are calculated, such as *q* and *d* from 2θ.^1^ If there are multiple diffraction data loops in a single data block, then they are copied into a single data block of their own, along with their relevant metadata, maintaining their relative ordering. Quantitative phase analysis results are collated from all phases and diffraction patterns, predicated on the _pd_phase_name uniquely identifying each phase present in all patterns. The various plotting menus are populated with values based on the CIF file contents, and sensible default plotting attributes are applied. The user is then free to interactively visualize their data. *pdCIFplotter* relies on *Matplotlib* (Hunter, 2007 ▸; Hunter & Droettboom, 2021 ▸), *NumPy* (Harris *et al.*, 2020 ▸; Oliphant, 2021 ▸), *PySimpleGui* (Driscoll, 2021 ▸) and *mplcursor* (Lee, 2021 ▸) and uses *PyInstaller* (Mandeljc *et al.*, 2021 ▸).

### 2.2. Single plot

**Figure 2:**
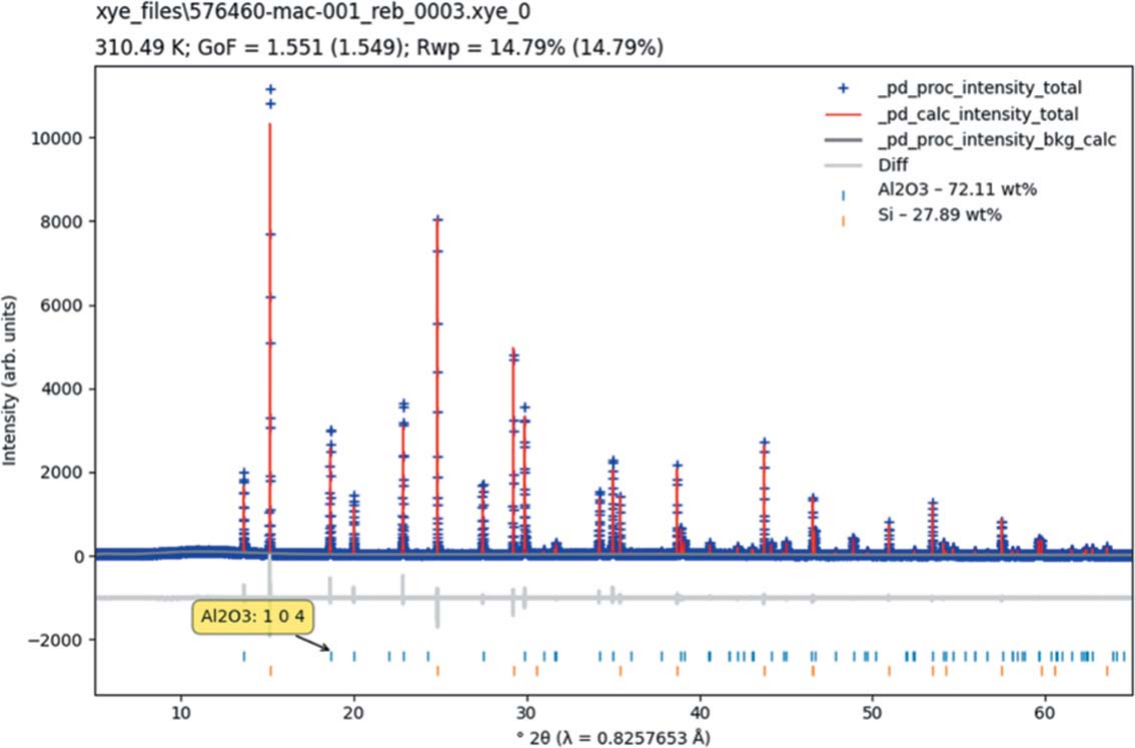
An example of a single diffraction pattern plot. The plot title is the block id. The subtitle summarizes the date/time, temperature, pressure, goodness of fit (GoF) and *R*_wp_ values, if given. If it is possible, the GoF and *R*_wp_ values are calculated and given in brackets. Quantitative phase results are displayed for each phase if given. Information about each HKL tick mark is given by hover text. Example data taken from Evans (2021*a* ▸).

**Figure 3:**
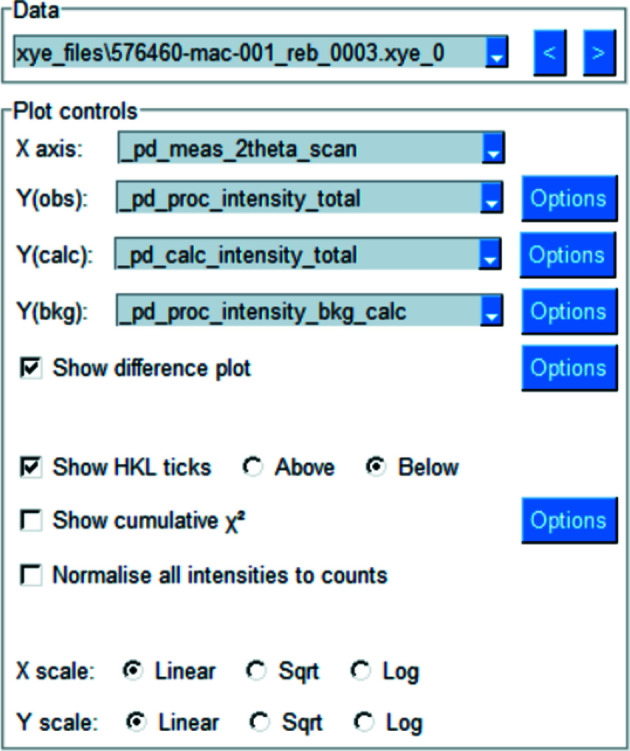
Single plot options. Different diffraction data can be chosen from the first drop-down box. The next or previous data set can be chosen with the arrows. The data to be plotted can be chosen with the next four drop-down boxes. Other plot options can be chosen with the checkmarks. The scale of each axis can be controlled independently. The colour and styling of each line can be set using the ‘Options’ button.

The single plot is designed for visualizing a single diffraction pattern at a time, most often in conjunction with the results of a Rietveld model (see Fig. 2 ▸ for an example use-case). Fig. 3 ▸ shows the options for controlling the data to be plotted. The block ids of the available data are shown in the first drop-down box. The *x* and *y* coordinates to be plotted can be chosen via the next four drop-down boxes (see Table 1 ▸ for the complete options). The contents of these boxes are dynamically updated from the available data as different source data are chosen. If the user does not wish to plot a certain data name, then ‘None’ can be chosen in the drop-down. If a certain data name is not available, then ‘None’ is displayed.

A difference plot, defined as *Y*(obs) − *Y*(calc), can be displayed. The difference plot is dynamically calculated and is automatically offset from the main diffraction pattern so as not to clash with the other displayed data. HKL ticks, or reflection markers, indicate the position of each reflection of each crystalline phase, as long as the phases have _ref ln_d_spacing defined and as long as it is possible to convert *d* spacing into the *x* ordinate of choice. By default, the tick marks are displayed below the diffraction pattern, with phases vertically offset from each other. The phase name and HKL information for a reflection are displayed when hovering the mouse cursor over the tick mark.

Cumulative χ^2^ is a function showing how the goodness of fit (Young, 1995 ▸) evolves across the diffraction pattern (David, 2004 ▸). Ideally, the function should be a smooth curve, with the presence of steps indicating regions of misfit between the model and data. To give an indication of the uncertainty associated with each data point, ‘Normalise all intensities to counts’ should be used. This calculates a normalization constant – *Y*(norm) = *Y*(obs)/*Y*(err)^2^ – to scale all displayed intensities, which has the effect of converting the given intensities to counts such that *y*/sqrt(*y*) = *Y*(obs)/*Y*(err). The end result is that the uncertainty of each data point is the square root of its displayed value.

The *x* and *y* axes can both be independently scaled to show data in linear, square root or log_10_ scales of the respective coordinate. If the chosen *x* coordinate is related to 2θ, the wavelength, if given, will be shown in the axis label.

The ‘Options’ buttons provide the ability to set the line/marker style, colour and size of the individual plot components.

**Figure 4:**
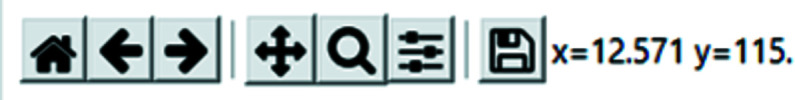
Plot navigation toolbar, showing the coordinates of the cursor on the plot.

The plot automatically shows a legend denoting each of the constituent parts. The colours for the HKL ticks are automatically assigned. The view in the plot window can be manipulated with the navigation toolbar (*Matplotlib*, 2020 ▸) shown in Fig. 4 ▸. The ‘Home’ button resets the view to the complete pattern, and the arrows move between previously defined views. The crossed arrows button activates pan. With this button activated, place the mouse cursor in the plot, click the left mouse button and drag the plot to a new position. The magnifying glass activates the zoom rectangle. With this button activated, place the mouse cursor in the plot, click the left mouse button and drag a rectangle to define a region to zoom into. After the mouse button is released, the region defined by the rectangle will be expanded to fill the plot. The pan and zoom behaviour can be modified by pressing the x, y or control keys. The (*x*, *y*) coordinates of the cursor in the plot are also shown dynamically in the toolbar.

### 2.3. Stack plot

**Figure 5:**
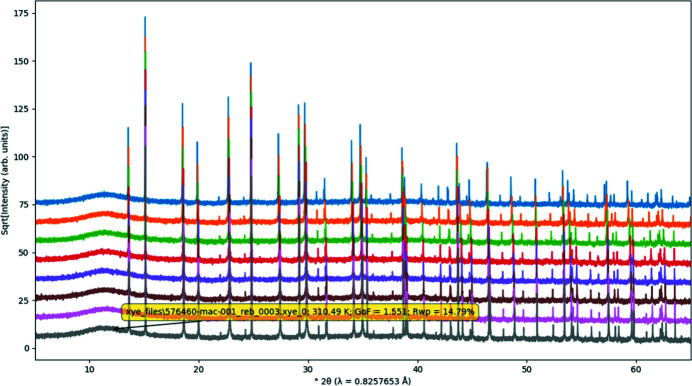
Example of a stack plot. Hover text for each diffraction pattern displays the block id, date/time, temperature, pressure, goodness of fit and *R*_wp_ values, if given. Example data taken from Evans (2021*a* ▸).

**Figure 6:**
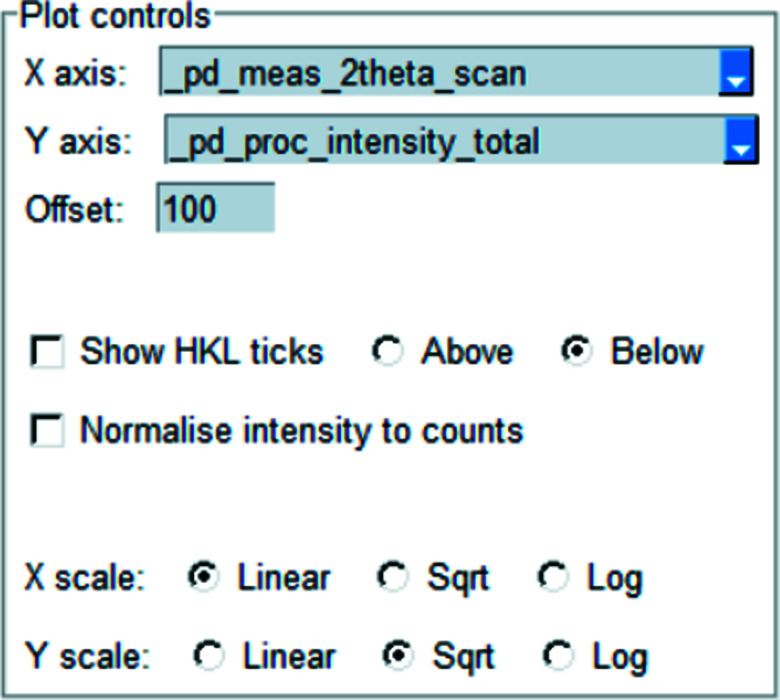
Stack plot options. The coordinates to plot can be chosen from the drop-down boxes. Other plot options can be chosen via the checkboxes and radio buttons. The scale of each axis can be controlled independently.

Stack plots are used to display multiple diffraction patterns where each pattern is vertically offset from the others (see Fig. 5 ▸ for an example). The first pattern to appear in the CIF file is the lowest pattern in the plot. The options available in the stack plot are shown in Fig. 6 ▸. The stack plot will show all patterns which have the combination of *x* and *y* coordinates given. The ‘X axis’ drop-down contains all possible *x* coordinates, and the ‘Y axis’ drop-down contains all possible *y* coordinates which also have the given *x* coordinate. The vertical offset can be altered using the given offset parameter. Individual patterns can be identified by hover text appearing when the mouse pointer hovers over the pattern. The colours of individual patterns are chosen automatically. HKL ticks can be shown, intensities can be normalized and axes scaled, as outlined in Section 2.2

### 2.4. Surface

**Figure 7:**
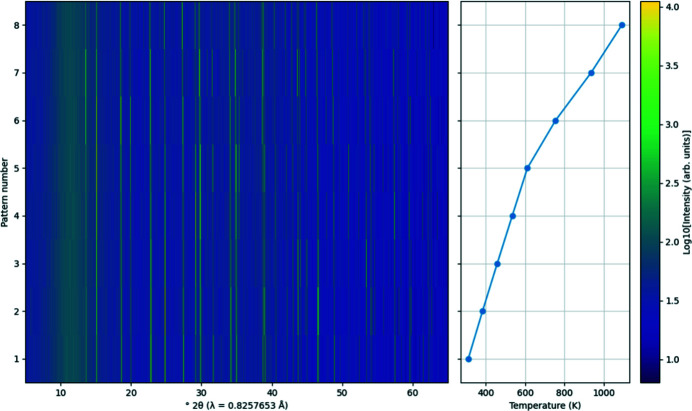
Example of a surface plot with the optional plot showing the temperature at which the diffraction data were collected. Note the common *y* axis between the two plots. Example data taken from Evans (2021*a* ▸).

**Figure 8:**
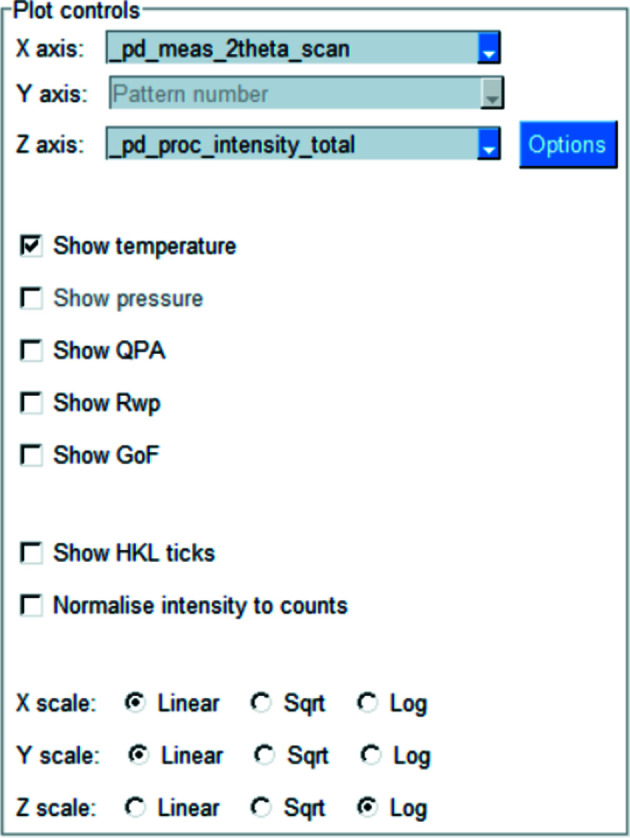
Surface plot options. The *x* and *z* coordinates can be chosen from the drop-down boxes, and the temperature, pressure, quantitative phase analysis, *R*_wp_ or GoF can be optionally shown in a secondary plot. The scale of each main plot axis can be controlled independently.

Surface plots show a series of diffraction patterns where the intensity of each point is represented by a colour (see Fig. 7 ▸ for an example). This type of plot is an alternative to stack plots and can highlight changes in unit-cell parameters or phase composition. Each diffraction pattern is linearly interpolated onto a common grid to allow for dissimilar patterns to be displayed together. Fig. 8 ▸ shows the plotting options. The ‘X axis’ drop-down contains all possible *x* coordinates, and the ‘Z axis’ drop-down contains all possible intensity data names which also have the given *x* coordinate. The ‘Y axis’ drop-down is fixed on pattern number. Changing the *z*-axis scale alters the colours assigned to each data point. HKL ticks can be shown, intensities can be normalized and axes scaled, as outlined in Section 2.2

## 3. Example data

Four example data sets are provided in the supplementary information to showcase *pdCIFplotter*’s capabilities. All CIF output is from the method outlined in Appendix *A* [App appa] using *TOPAS* v7.

(1) A CIF file compiled from Rietveld refinement of synchrotron temperature calibration data (Evans, 2021*a* ▸).

(2) A CIF file compiled from Rietveld refinement of a laboratory variable-temperature data set (Billing, 2022 ▸).

(3) A CIF file compiled from Rietveld refinement of multiple-detector-bank time-of-flight (TOF) neutron data (Ainsworth *et al.*, 2015 ▸; Evans, 2021*b* ▸).

(4) A CIF file compiled from Rietveld refinement of an *in situ* multi-phase synchrotron data set.

## 4. Program availability

*pdCIFplotter* is written in Python 3 and is available for download and installation from github (https://github.com/rowlesmr/pdCIFplotter) and PyPI (https://pypi.org/project/pdCIFplotter/). The program is made available under the Apache 2.0 licence. For ease of use, *pdCIFplotter* can be downloaded as a zip file containing all necessary Windows files (https://www.iucr.org/__data/iucr/powder/pdcif_apps/pdCIFplotter.zip; https://www.iucr.org/resources/commissions/powder-diffraction/projects/pdcif/pdcifplotter). If you do wish to install from source, installation instructions are available on the github page. Installation via *pip* (> pip install pdcifplotter) will automatically download and install all required dependencies. After installation, the program can be started from the command line with the command pdcifplotter, or a shortcut can be made in your desktop environment. Currently, Windows installation of the key dependency *PyCifRW* requires Microsoft Visual C++ 14.0 or greater; Linux and Mac operating systems should already have the required compiler. If you do not have the compiler or do not wish to install it, third-party precompiled files are available (Gohlke, 2021 ▸).

## Supplementary Material

cif.inc - TOPAS include file for creating powder CIF files. DOI: 10.1107/S1600576722003478/yr5087sup1.zip

First example - ZIP file holding CIF file, TOPAS input file and diffraction data. DOI: 10.1107/S1600576722003478/yr5087sup2.zip

Second example - ZIP file holding CIF file, TOPAS input file and diffraction data. DOI: 10.1107/S1600576722003478/yr5087sup3.zip

Third example - ZIP file holding CIF file, TOPAS input file and diffraction data. DOI: 10.1107/S1600576722003478/yr5087sup4.zip

Fourth example - ZIP file holding CIF file, TOPAS input file and diffraction data. DOI: 10.1107/S1600576722003478/yr5087sup5.zip

Mini examples on some possible ways to use the pdCIF macros in cif.inc with TOPAS. DOI: 10.1107/S1600576722003478/yr5087sup6.zip

Information on how to download the program. DOI: 10.1107/S1600576722003478/yr5087sup7.txt

**Figure 9:**
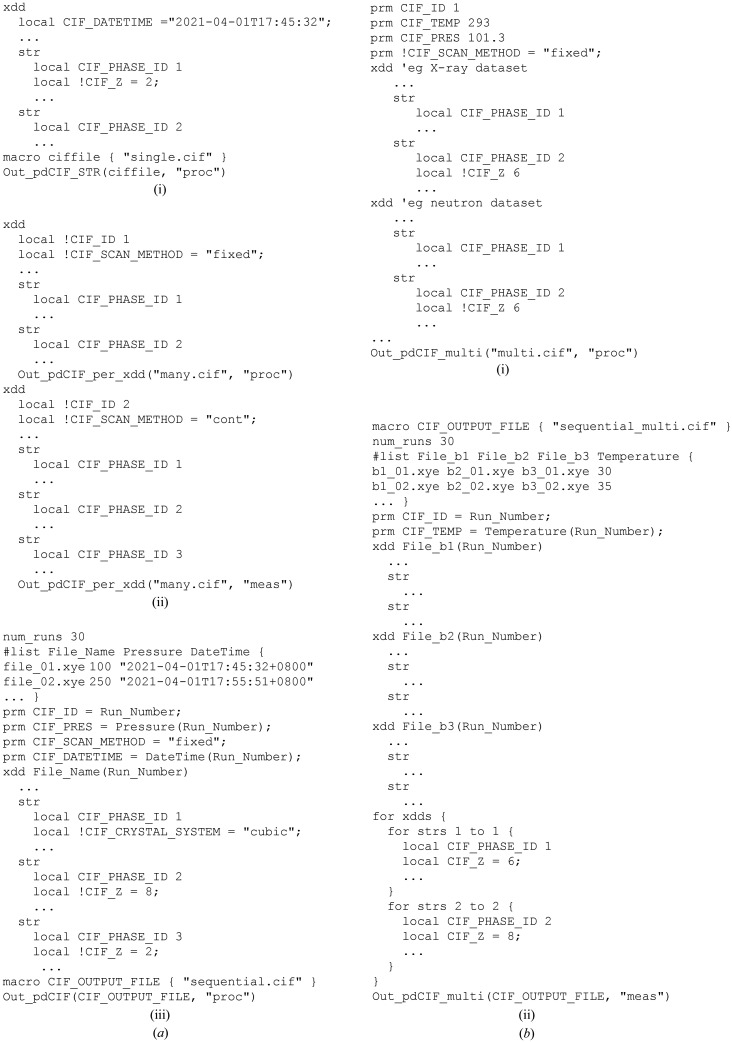
Example uses of the CIF output macros. (*a*) Out_pdCIF: (i) Simple, single diffraction pattern refinement with one crystal structure of interest and an impurity. (ii) Fitting data from two different synthesis methods, with each containing a different number of structures. (iii) Sequential refinement of *in situ* diffraction data. (*b*) Out_pdCIF_multi: (i) Combined refinement of two crystal structures using X-ray and neutron data. (ii) Sequential refinement of *in situ* diffraction data with two crystal structures across three diffraction patterns.

PDF version of Fig. 9. DOI: 10.1107/S1600576722003478/yr5087sup8.pdf

## Figures and Tables

**Table 1:** pdCIF data names associated with diffraction intensities and their position

*X* coordinates	Observed intensities *Y*(obs)	Intensity uncertainty *Y*(err)	Calculated intensities *Y*(calc)	Background intensities *Y*(bkg)
_pd_meas_2theta_scan	_pd_meas_counts_total	_pd_meas_counts_total †	_pd_calc_intensity_total	_pd_meas_counts_background
_pd_proc_2theta_corrected	_pd_meas_intensity_total	_pd_meas_intensity_total ‡	_pd_calc_intensity_net	_pd_meas_counts_container
_pd_meas_time_of_f light	_pd_proc_intensity_total	_pd_proc_intensity_total ‡		_pd_meas_intensity_background
_pd_meas_position	_pd_proc_intensity_net	_pd_proc_intensity_net ‡		_pd_meas_intensity_container
_pd_proc_energy_incident		_pd_proc_ls_weight §		_pd_proc_intensity_bkg_calc
_pd_proc_d_spacing				_pd_proc_intensity_bkg_f ix
_pd_proc_recip_len_Q				
_pd_meas_2theta_range_ ¶				
_pd_proc_2theta_range_ ¶				

† The uncertainty is given as the square root of the intensity.

‡ The uncertainty must be explicitly given, or given as _pd_proc_ls_weight.

§ The uncertainty is the inverse square root of this value.

¶ These correspond to three different data items: ###_min, ###_max and ###_inc, denoting the minimum and maximum diffraction angle and the equidistant step size between them.

**Table 2:** Other pdCIF and CIF data names that provide useful metadata for *pdCIFplotter*

_pd_phase_name
_dif frn_radiation_wavelength
_dif frn_ambient_temperature
_dif frn_ambient_pressure
_pd_phase_mass_%
_pd_meas_datetime_initiated
_ref ine_ls_goodness_of_f it_all
_pd_proc_ls_prof_wR_factor

**Table 3:** Predefined parameter names for the Out_pdCIF and Out_pdCIF_multi macros

Parameter name	Scope†	Description
CIF_ID ‡	xdd	Unique identifier for each diffraction pattern
CIF_PHASE_ID ‡	str	Unique identifier for each structure
CIF_SCAN_METHOD §	xdd	How intensities were scanned when collected
CIF_DATETIME	xdd	Date/time when data collection was initiated
CIF_TEMP	xdd	The temperature (K) at which the data were collected
CIF_PRES	xdd	The pressure (kPa) at which the data were collected
CIF_Z	str	Formula units per unit cell
CIF_SUM_FORMULA	str	Chemical formula of the unit cell
CIF_CRYSTAL_SYSTEM	str	Crystal system of the structure
CIF_t0, CIF_t1, CIF_t2 ‡	xdd	Time-of-flight calibration constants
CIF_TH2_F IXED ‡	xdd	Detector angle for energy-dispersive data
CIF_DIF FRACTOGRAM_BLOCK_ID ¶	xdd	Fully specified block ID for a diffraction pattern
CIF_PHASE_BLOCK_ID ¶	str	Fully specified block ID for a crystal structure
CIF_DIFFRACTOGRAM_TEXT	xdd	Free-text entry in the data block containing diffraction data
CIF_PHASE_TEXT	str	Free-text entry in the data block containing structure data

† ‘xdd’ is the *TOPAS* keyword meaning X-ray diffraction data. In an input file, the xdd scope contains the diffraction data and all crystal structures relating to that diffraction data. ‘str’ is the *TOPAS* keyword meaning ‘crystal structure’. In an input file, the str scope contains all crystal and micro-structural parameters for a single crystallographic phase.

‡ Must have values set.

§ Takes the values "step", "cont" or "fixed" for step scan, continuous scan or stationary detector, respectively. Should be set for constant-wavelength data.

¶ Will override any automatically generated block IDs.

## Footnotes

1 This conversion simply calculates the new *x* coordinate in terms of the old. No attempt is made to resolve multiple peaks caused by multiple wavelengths.

## Appendix A

In testing *pdCIFplotter*, a series of macros were developed for the production of CIF files from Rietveld refinements in *TOPAS* (Coelho, 2018 ▸). These macros are available in the supplementary information and on the *TOPAS* wiki (Evans, 2010 ▸; http://topas.dur.ac.uk/topaswiki/doku.php?id=out_pdcif). They have been tested in *TOPAS* v5–7, and have been designed to work with constant-wavelength X-ray and neutron diffraction data, TOF neutron diffraction data, and energy-dispersive X-ray diffraction (EDXRD) data. Two main user-facing macros have been developed, each representing a different refinement use-case:

(1) Each diffraction pattern in a single input file is independent of the other patterns. Each diffraction pattern can be modelled with a different number of structures. For example, a single laboratory diffraction pattern, or an *in situ* temperature series where a single temperature is represented by a single diffraction pattern. These are ‘single’ refinements.

(2) Diffraction patterns in a single input file form groups, where each diffraction pattern in each group is modelled by a common set of structures. For example, multiple-detector-bank TOF diffraction data, or a combined refinement of X-ray and neutron data. These are ‘multi’ refinements.

The key macros are (i) Out_pdCIF(cif f ile, data_type, bkg_eqn) and (ii) Out_pdCIF_multi(cif f ile, data_type, bkg_eqn), which write both crystal structure and powder diffraction data to file. cif f ile is the name of the file to which the information is appended. data_type is either "proc" or "meas" and represents data that have been processed, for example, combination of diffraction patterns, corrections for background, detector dead time *etc*., or represents data that are as-measured and have had no alterations made, respectively. bkg_eqn is the equation describing the background so that it can be given in the CIF file. Helper macros are provided which give sensible defaults for the second and third macro arguments if only the first or first and second arguments are given. See the mini examples and cif.inc in the supplementary information for further information and implementation details.

Depending on the analysis being undertaken, certain variables must, should or could be set using pre-defined names (see Table 3 ▸). Each xdd or group of xdds must be given a different identifier which is used as part of the block ID of each constituent structure and diffraction pattern to ensure that it is unique.^2^ Each structure must also be given a unique identifier to assist in the generation of a block ID and to number each phase in the HKL lists. The method by which the detector was scanned when collecting data should be given: "step", "cont" or "fixed" for step scan, continuous scan or stationary detector, respectively (this is automatically set for TOF or EDXRD data). The date and time at which data collection was initiated should be specified. If the diffraction data were collected under non-ambient conditions, then the temperature (in K) and/or pressure (in kPa) must be defined in each xdd. The number of formula units per unit cell can be set in each structure if the user wishes to output the molecular weight. The sum formula and crystal system of each structure can also be specified. If time-of-flight neutron data are being analysed, then the three calibration constants, such that TOF = *t*_0_ + *t*_1_ *d* + *t*_2_ *d* ^2^, must be given. If energy-dispersive data are being analysed, the 2θ angle, in degrees, of the detector must be given. It is also possible to fully specify the block ID for diffraction patterns and structures if you do not wish to use the automatically generated IDs.

Some examples of how these macros could be used are given in Fig. 9 ▸. The filename should have no spaces, each structure must have a phase name and space group defined with no spaces. In each case, underscores can be used in lieu of spaces. If the same phase appears in more than one diffraction pattern then the same phase name should be used.
