# Supplementary material for: pdCIFplotter: visualizing powder diffraction data in pdCIF format
Source: J Appl Crystallogr. 2022 May 25;55(Pt 3):631–7. doi: 10.1107/S1600576722003478 (PMC9172038; doi:10.1107/S1600576722003478)
Supplement: Supplementary file 8 [file j-55-00631-sup8.pdf]

# pdCIFplotter: visualizing powder diffraction data in pdCIF format

```
xdd
  local CIF_DATETIME ="2021-04-01T17:45:32";
  ...
  str
    local CIF_PHASE_ID 1
    local !CIF_Z = 2;
    ...
  str
    local CIF_PHASE_ID 2
    ...
macro ciffile { "single.cif" }
Out_pdCIF_STR(ciffile, "proc")
```

(i)

```
xdd
  local !CIF_ID 1
  local !CIF_SCAN_METHOD = "fixed";
  ...
  str
    local CIF_PHASE_ID 1
    ...
  str
    local CIF_PHASE_ID 2
    ...
  Out_pdCIF_per_xdd("many.cif", "proc")
xdd
  local !CIF_ID 2
  local !CIF_SCAN_METHOD = "cont";
  ...
  str
    local CIF_PHASE_ID 1
    ...
  str
    local CIF_PHASE_ID 2
    ...
  str
    local CIF_PHASE_ID 3
    ...
  Out_pdCIF_per_xdd("many.cif", "meas")
```

(ii)

```
num_runs 30
#list File_Name Pressure DateTime {
file_01.xye         100 "2021-04-
01T17:45:32+0800"
file_02.xye         250 "2021-04-
01T17:55:51+0800"
... }
prm CIF_ID = Run_Number;
prm CIF_PRES = Pressure(Run_Number);
prm CIF_SCAN_METHOD = "fixed";
prm CIF_DATETIME = DateTime(Run_Number);
xdd File_Name(Run_Number)
  ...
  str
    local CIF_PHASE_ID 1
    local !CIF_CRYSTAL_SYSTEM = "cubic";
    ...
  str
    local CIF_PHASE_ID 2
    local !CIF_Z = 8;
    ...
  str
    local CIF_PHASE_ID 3
    local !CIF_Z = 2;
     ...
macro CIF_OUTPUT_FILE { "sequential.cif" }
Out_pdCIF(CIF_OUTPUT_FILE, "proc")
```

(iii)

(*a*)

```
prm CIF_ID 1
prm CIF_TEMP 293
prm CIF_PRES 101.3
prm !CIF_SCAN_METHOD = "fixed";
xdd 'eg X-ray dataset
   ...
   str
      local CIF_PHASE_ID 1
      ...
   str
      local CIF_PHASE_ID 2
      local !CIF_Z 6
      ...
xdd 'eg neutron dataset
   ...
   str
      local CIF_PHASE_ID 1
      ...
   str
      local CIF_PHASE_ID 2
      local !CIF_Z 6
      ...
...
Out_pdCIF_multi("multi.cif", "proc")
```

(i)

```
macro CIF_OUTPUT_FILE { "sequential_multi.cif" }
num_runs 30
#list File_b1 File_b2 File_b3 Temperature {
b1_01.xye b2_01.xye b3_01.xye 30
b1_02.xye b2_02.xye b3_02.xye 35
... }
prm CIF_ID = Run_Number;
prm CIF_TEMP = Temperature(Run_Number);
xdd File_b1(Run_Number)
  ...
  str
    ...
  str
    ...
xdd File_b2(Run_Number)
  ...
  str
    ...
  str
    ...
xdd File_b3(Run_Number)
  ...
  str
    ...
  str
    ...
for xdds {
  for strs 1 to 1 {
    local CIF_PHASE_ID 1
    local CIF_Z = 6;
    ...
  }
  for strs 2 to 2 {
    local CIF_PHASE_ID 2
    local CIF_Z = 8;
    ...
  }
}
Out_pdCIF_multi(CIF_OUTPUT_FILE, "meas")
```

(ii)

(*b*)

Example uses of the CIF output macros. (*a*) `Out_pdCIF`: (i) Simple, single diffraction pattern refinement with one crystal structure of interest and an impurity. (ii) Fitting data from two different synthesis methods, with each containing a different number of structures. (iii) Sequential refinement of in situ diffraction data. (*b*) `Out_pdCIF_multi`: (i) Combined refinement of two crystal structures using X-ray and neutron data. (ii) Sequential refinement of in situ diffraction data with two crystal structures across three diffraction patterns

Matthew Rowles
